# The relationship between fermented and nonfermented dairy products consumption and hypertension among premature coronary artery disease patients: Iran premature coronary artery disease study

**DOI:** 10.1002/fsn3.3998

**Published:** 2024-02-08

**Authors:** Shakila Ansari, Noushin Mohammadifard, Parisa Hajihashemi, Fahimeh Haghighatdoost, Ehsan Zarepur, Shirin Mahmoudi, Fatemeh Nouri, Fereydoon Nouhi, Tooba Kazemi, Nahid Salehi, Kamal Solati, Samad Ghaffari, Mahboobeh Gholipour, Mostafa Dehghani, Mostafa Cheraghi, Habib Heybar, Hassan Alikhasi, Nizal Sarrafzadegan

**Affiliations:** ^1^ Department of Community Nutrition, Nutrition and Food Security Research Center, School of Nutrition and Food Science Isfahan University of Medical Sciences Isfahan Iran; ^2^ Interventional Cardiology Research Center, Cardiovascular Research Institute Isfahan University of Medical Sciences Isfahan Iran; ^3^ Isfahan Gastroenterology and Hepatology Research Center Isfahan University of Medical Sciences Isfahan Iran; ^4^ Isfahan Cardiovascular Research Center, Cardiovascular Research Institute Isfahan University of Medical Sciences Isfahan Iran; ^5^ Department of Cardiology, Medicine School Isfahan University of Medical Sciences Isfahan Iran; ^6^ Rajaie Cardiovascular Medical and Research Center Iran University of Medical Sciences Tehran Iran; ^7^ Iranian Network of Cardiovascular Research (INCVR) Tehran Iran; ^8^ Cardiovascular Diseases Research Center Birjand University of Medical Sciences Birjand Iran; ^9^ Cardiovascular Research Center, Health Institute Kermanshah University of Medical Sciences Kermanshah Iran; ^10^ Department of Psychiatry Shahrekord University of Medical Sciences Shahrekord Iran; ^11^ Cardiovascular Research Center Tabriz University of Medical sciences Tabriz Iran; ^12^ Department of Cardiology, Healthy Heart Research Center, Heshmat Hospital, School of Medicine Guilan University of Medical Sciences Rasht Iran; ^13^ Department of Cardiovascular Research Center, Shahid Rahimi Hospital Lorestan University of Medical Sciences Khorramabad Iran; ^14^ Atherosclerosis Research Center Ahvaz Jundishapur University of Medical Sciences Ahvaz Iran; ^15^ Heart Failure Research Center, Cardiovascular Research Institute Isfahan University of Medical Sciences Isfahan Iran; ^16^ Faculty of Medicine, School of Population and Public Health University of British Columbia Vancouver Canada

**Keywords:** dairy product, fermented dairy product, fermented milk products, hypertension, The Food and Agriculture Organization of the United Nations

## Abstract

Dairy products may affect hypertension (HTN) risk. The aim of this study was to examine the association between fermented and nonfermented dairy foods and HTN in a sample of premature coronary artery disease (PCAD) subjects. This cross‐sectional study was performed on 1854 PCAD patients. A 110‐item food frequency questionnaire was used to assess dietary intakes. HTN was considered if systolic blood pressure was 140 mmHg and higher and/or diastolic blood pressure was 90 mmHg and higher. The odds ratio of HTN across the quartiles of different types of dairy products was evaluated by binary logistic regression. The mean (SD) of dairy products consumption was 339.8 (223.5) g/day, of which 285.4 g/day was fermented dairy products. In the crude model, participants in the fourth quartile of fermented dairy products had lesser risk of HTN compared to the bottom quartile (OR = 0.70, 95% CI: 0.52, 0.96; *p* for trend = .058). However, after considering the possible confounders, the significance disappeared. Subjects in the top quartile of high‐fat fermented dairy products had 34% lower risk for HTN compared to the bottom quartile (95% CI: 0.49, 0.88; *p* for trend < .001). Adjustment for potential risk factors weakened the association but remained significant (OR = 0.73, 95% CI: 0.53, 1.01; *p* for trend = .001). Nonsignificant relation was detected between low‐fat fermented, low‐fat nonfermented, and high‐fat nonfermented dairy products and HTN. Moderate consumption of high‐fat fermented dairy products, in a population with low consumption of dairy foods, might relate to reduced likelihood of HTN.

## Graphical abstract

A cross‐sectional study was conducted on 1854 premature coronary artery disease patients. Participants with higher intake of fermented dairy products had lower risk of hypertension. Subjects with higher intake of high‐fat fermented dairy products had lower risk of hypertension. No significant association was found between low‐fat fermented, low‐fat nonfermented, and high‐fat nonfermented dairy products and hypertension.

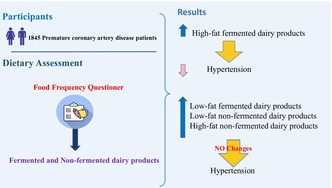

## 1 INTRODUCTION

Hypertension (HTN) is considered to be an essential public health problem globally. In 2021, the new guideline for the pharmacological treatment of HTN published by the World Health Organization (WHO) showed that the worldwide occurrence of HTN was approximately 1.4 billion people (Organization, 2022). The prevalence of HTN is also relatively high in Iran, and based on the results of a meta‐analysis, it is estimated to be around 25% (Oori et al., 2019). HTN is narrowly linked to coronary artery disease. It is assumed that HTN is one of the underlying causes of premature coronary artery disease (PCAD) (Poorzand et al., 2019). With a 10 mmHg increase in systolic blood pressure (SBP), the possibility of stroke and fatal myocardial infarction raised by 53% and 31%, respectively, in Asian population (Che et al., 2013).

Dietary modification can play a vital part in anticipation and control of HTN. Dairy products, such as milk and yogurt, are good sources of potassium, calcium, and proteins of high biological value (Mirmiran et al., 2016). These properties make dairy consumption as a protective way against many chronic disease such as diabetes, metabolic syndrome (Bhavadharini et al., 2020), osteoporosis (Ansari et al., 2023), and HTN (Feng et al., 2022) The Dietary Approaches to Stop HTN (DASH) is the most successful dietary pattern observed to minor blood pressure (BP) (Filippou et al., 2020). The advantageous impact of DASH diet on HTN seems to be more than vegetarian patterns (Appel et al., 2006), recommending a probable impact of other components such as dairy products. Dairy foods have inhibitory peptides called angiotensin I‐converting enzyme (ACE), which are supposed to be practical in the prevention of HTN (Herregods et al., 2011).

Previous studies have proposed calcium as the reason for the antihypertensive effect of dairy products (McGrane et al., 2011; Wang et al., 2008). Nevertheless, the association between dairy consumption and HTN is inconclusive. A prospective cohort study on the middle‐aged US women stated that low‐fat dairy foods were inversely associated with HTN risk (Wang et al., 2008). Additionally, Toledo et al. (2008) revealed that dairy intake was in line with lower blood pressure in older people. In contrast, a cohort study revealed nonsignificant relation between 5‐year odds of HTN and dairy consumption (Dauchet et al., 2007). A prospective cohort study on normotensive adults reported that women with greater intakes of total and semiskimmed dairy were inversely related to the 3‐year occurrence of HTN, however greater total dairy intakes augmented the risk of HTN in men (Mirmiran et al., 2016). Not only does fat content of dairy products determine health benefit of dairy products, but also manufacturing process, such as fermentation, has a key role to play. The fermentation procedure involves adding beneficial bacteria to dairy products which have antihypertensive properties. For instance, adding *Lactobacillus* to dairy foods can produce angiotensin‐converting enzyme (ACE) inhibitory peptides (Chen et al., 2021; Koskinen et al., 2018). These peptides exert an inhibitory role, thereby prevent the transformation of angiotensin I to angiotensin II, which results in a decrease in blood pressure (Liu et al., 2012). Results of a randomized controlled trial declared that consuming 150 mL of fermented milk twice a day had BP‐lowering effect on hypertensive subjects (Jauhiainen et al., 2005).

Despite numerous studies conducted on the correlation between fermented and nonfermented dairy products and HTN, up to now, there has been no research conducted on PCAD participants. Moreover, variations in lifestyle, dietary preferences, the average intake of dairy products within different populations, and diverse fermentation techniques can lead to conflicting findings across various research studies. Hence, the chief aim of this study is to assess the correlation between fermented and nonfermented dairy foods and HTN in a multicentric study of PCAD subjects.

## 2 METHODS

### 2.1 Study population

This cross‐sectional study was conducted based on the Iran premature coronary artery disease (IPAD) study data, an ongoing multicentric study that focuses on Iranian patients with diverse ethnic backgrounds. Additional information regarding the study's design and sampling method has been presented elsewhere (Zarepur et al., 2020). This study was performed on PCAD patients, which were recruited from 15 cities based on the distribution of races (Fars, Azari, Arab, Lor, Gilak, Balouch, Turkaman, Qashqai, and Bakhtiari). PCAD was defined as a 75% or more severe stenosis in at least one coronary artery or a 50% or more severe stenosis in the left main coronary artery. The following inclusion criteria were applied: (1) taking coronary angiography, (2) women <70 years old and men <60 years old, (3) be one of the desired ethnic groups, and (4) know their parent's ethnicity. Individuals who have been diagnosed with coronary artery disease, such as those who have undergone procedures like balloon angioplasty, coronary artery bypass grafting, or percutaneous coronary intervention were removed from this study. A total of 1854 individuals were considered to be included in statistical analysis. The written informed consent, which received approval from the Ethics Committee of Isfahan University of Medical Sciences (IR.ARI.MUI.REC.1402.112), was provided by all the participants who registered for the study.

### 2.2 Data collection

In each city with a major ethnic group, major cardiac catheterization centers were selected and requested to employee persons who met the recruitment criteria. The participants were requested to contribute in the study after explaining the research content and obtaining their consent. Data on demographic characteristics such as age, gender, ethnicity, religion, salary, education, and marital status were provided and recorded by qualified interviewers. A validated questionnaire was taken to evaluate lifestyle factors such as smoking, alcohol or drug consumption, and physical activity. Anthropometric measurements, such as weight, height, and waist circumference, were evaluated by trained personnel using the normal protocols. The calculation of body mass index (BMI) entails dividing an individual's weight in kilograms by the square of their height in meters. Physical activity level was measured through the use of the International Physical Activity Questionnaire–Short Form (IPAQ‐SF). Based on this questionnaire, an individual's physical activity level was classified as “low,” “moderate,” or “high.” The participants' blood pressure was measured by the use of a digital sphygmomanometer with the appropriate cuff using the standard method. HTN was defined as having a systolic blood pressure (SBP) of 140 mmHg or higher or a diastolic blood pressure (DBP) of 90 mmHg or higher. Blood samples were obtained after 12 h overnight fasting, and examined for fasting blood sugar (FBS), high‐density lipoprotein cholesterol (HDL‐c), low‐density lipoprotein cholesterol (LDL‐c), triglyceride (TG), and total cholesterol (TC).

### 2.3 Dietary assessment

Regular dietary intake in the preceding year was evaluated using a validated 110‐item, semiquantitative food frequency questionnaire (FFQ) (Mohammadifard et al., 2021). The determination of portion size for each food item was according to a common serving size. Participants were requested to indicate the frequency at which they consumed each food using nine multiple response categories ranging from not once/rarely to more than 6 times per day. Participants' daily intake of each food item was calculated and converted to grams per day, taking into account the weight of each portion and the frequency of consumption. The daily energy and nutrient intake were then estimated using the Nutritionist IV software modified for Iranian cuisine. Dough, cheese, yogurt, and curd were considered in the category of fermented dairy products and milk was considered in the category of nonfermented dairy products.

### 2.4 Statistical analysis

Baseline characteristics of PCAD participants were compared across the quartile of fermented and nonfermented dairy product intake using one‐way analysis of variance (ANOVA) for continuous variables and chi‐square test for categorical variables. Continuous and categorical variables were reported as mean ± standard and frequency (*n*, %), respectively. To assess the association between PCAD and quartiles of fermented and nonfermented dairy product intake, logistic regression of the crude and adjusted models was performed. Crude and multivariable‐adjusted odds ratios (OR) and 95% confidence intervals (CIs) as the association measures were calculated. The lowest quartile of fermented and nonfermented dairy products intake was considered reference category. In the first adjusted model, the confounding effects of age (years), sex (male/female), and daily energy intake (kcal/day) were controlled. In the second model, further adjustment for smoking (yes/no), level of education (under graduate/graduate/postgraduate), ethnicity, and marital status (single/married/divorce or widow) was made, and the third model was further adjusted for BMI (kg/m^2^). All statistical analyses were performed utilizing SPSS Statistics 26.0 (IBM, Armonk, NY). A significance level of *p* < .05 was deemed statistically significant for all statistical analyses.

## 3 RESULTS

This study encompassed a total of 1854 PCAD patients, of which 1219 (65.74%) were men. Mean age and BMI of the participants was 54.7 ± 7.1 years and 28.0 ± 4.5 kg/m^2^, respectively.

Table 1 depicts general characteristics of participants across different quartiles of fermented and nonfermented dairy products intake. Compared with those in the first quartile, those in the fourth quartile of fermented dairy intake were more likely to be current smoker, physically active, and had lower DBP. In terms of nonfermented dairy intake, subjects in the top quartile were more likely to be older and highly educated, but less likely to be men, current smoker, and physically active. However, SBP, DBP, and TG were significantly different across the quartiles of nonfermented dairy intake, with no specific trend. In both categories, the distribution of ethnicity varied across quartiles.

**TABLE 1:** General and metabolic characteristics of participants according to the quartile of fermented and nonfermented dairy products intake.

Intake quartile (range, g/day)	Fermented dairy products				*p* ^a^	Nonfermented dairy products				*p* ^a^
	Q1 (<397.0)	Q2 (397.0, 512.3)	Q3 (513.1, 653.9)	Q4 (>654.7)		Q1 (≤2.0)	Q2 (2.0, 16.6)	Q3 (16.6, 67.7)	Q4 (≥68.3)	
Subjects (*n*)	392	456	508	498	–	456	445	478	475	–
Sex (men), *n* (%)	250 (63.8)	301 (66.0)	343 (67.5)	325 (65.3)	.693	342 (75.0)	281 (63.1)	280 (58.6)	316 (66.5)	<.001
Age (years)	54.8 ± 7.3	54.9 ± 7.0	54.8 ± 6.9	54.3 ± 7.2	.547	53.8 ± 7.1	54.5 ± 7.3	55.6 ± 7.1	54.9 ± 6.8	.002
BMI (kg/m^2^)	27.8 ± 4.4	28.1 ± 4.4	27.8 ± 4.6	28.1 ± 4.6	.544	28.3 ± 4.4	28.0 ± 4.5	27.9 ± 4.6	27.7 ± 4.5	.208
Married, *n* (%)	354 (90.3)	410 (89.9)	470 (92.5)	450 (90.4)	.482	423 (92.8)	398 (89.4)	428 (89.5)	435 (91.6)	.225
Ethnicity, *n* (%)										
Fars	250 (63.9)	275 (60.3)	255 (50.5)	253 (50.8)	<.001	211 (46.4)	246 (55.3)	299 (62.7)	277 (58.6)	<.001
Azari	32 (8.2)	46 (10.1)	62 (12.3)	30 (6.0)		55 (12.1)	47(10.6)	32 (6.7)	36 (7.6)	
Kurd	25 (6.4)	41 (9.0)	58 (11.5)	57 (11.4)		69 (15.2)	46 (10.3)	31 (6.5)	35 (7.4)	
Lor and Bakhtiari	44 (11.3)	42 (9.2)	56 (11.1)	74 (14.9)		61 (13.4)	50 (11.2)	40 (8.4)	65 (13.7)	
Qashqaei and Arab and Balouch	12 (3.1)	23 (5.0)	23 (4.6)	34 (6.8)		22 (4.8)	24 (5.4)	24 (5.0)	22 (4.7)	
Gilak	28 (7.2)	29 (6.4)	51 (10.1)	50 (10.0)		37 (8.1)	32 (7.2)	51 (10.7)	38 (8.0)	
Education level, *n* (%)										
Undergraduate	185 (47.2)	219 (48.0)	243 (47.8)	251 (50.4)	.825	197 (43.2)	221 (49.7)	251 (52.5)	229 (48.2)	.045
Graduate	155 (39.5)	182 (39.9)	197 (38.8)	195 (39.2)		195 (42.8)	180 (40.4)	176 (36.8)	178 (37.5)	
Postgraduate	52 (13.3)	55 (12.1)	68 (13.4)	52 (10.4)		64 (14)	44 (9.9)	51 (10.7)	68 (14.3)	
Current smoking, *n* (%)	92 (23.5)	90 (19.7)	147 (28.9)	134 (26.9)	.006	139 (30.5)	110 (24.7)	90 (18.8)	124 (26.1)	.001
Physical activity	1328.1 ± 1752.7	1573.1 ± 2389.1	1612.1 ± 2322.7	1630.8 ± 2373.8	.002	1881.4 ± 2852.9	1540.9 ± 2135.9	1288.1 ± 1767.1	1819.3 ± 2564.8	<.001
Family history of CVD, *n* (%)	210 (55.4)	244 (55.2)	284 (58.1)	244 (50.0)	.081	234 (54.3)	249 (57.5)	251 (53.4)	248 (53.4)	.570
High WC (cm)	99.3 ± 11.0	100.6 ± 11.6	99.4 ± 11.5	99.2 ± 11.9	.237	100.2 ± 11.0	99.8 ± 12.3	99.0 ± 11.4	88.4 ± 11.5	.457
Hypertension, *n* (%)	106 (27.0)	103 (22.6)	123 (24.2)	103 (20.7)	.152	94 (20.6)	99 (22.2)	129 (27.0)	113 (23.8)	.124
Dyslipidemia, *n* (%)	282 (74.8)	336 (78.7)	377 (75.5)	378 (79.1)	.430	323 (77.3)	330 (79.1)	361 (78.3)	359 (77.0)	.871
Hypercholesterolemia, *n* (%)	55 (14.5)	66 (15.3)	67 (14.0)	73 (15.3)	.923	54 (12.9)	74 (17.7)	61 (13.1)	72 (15.5)	.167
Elevated LDL, *n* (%)	28 (7.4)	38 (8.9)	45 (9.4)	45 (9.5)	.713	34 (8.2)	37 (8.9)	38 (8.2)	47 (10.1)	.712
Low HDL, *n* (%)	216 (57.1)	246 (57.5)	294 (61.3)	284 (59.5)	.570	235 (56.4)	258 (62.0)	271 (58.5)	276 (59.1)	.421
Hypertriglyceridemia, *n* (%)	147 (39.2)	196 (45.9)	217 (45.5)	195 (40.9)	.124	183 (43.9)	186 (44.7)	194 (42.2)	192(41.5)	.753
FBS (mg/dL)	126.4 ± 63.7	122.7 ± 51.2	118.6 ± 58.0	117.5 ± 50.6	.111	115.1 ± 49.1	122.1 ± 56.9	121.7 ± 55.8	124.4 ± 60.3	.104
SBP (mmHg)	124.4 ± 18.9	122.1 ± 19.6	122.7 ± 19.2	121.2 ± 18.3	.087	120.4 ± 18.9	123.6 ± 17.5	124.1 ± 19.3	121.9 ± 20.1	.011
DBP (mmHg)	79.6 ± 11.6	77.7 ± 11.6	78.3 ± 11.9	76.8 ± 11.8	.005	77.2 ± 11.3	78.9 ± 11.5	78.6 ± 11.6	77.3 ± 12.5	.057
TC (mg/dL)	156.4 ± 43.0	158.8 ± 42.5	156.6 ± 45.3	159.2 ± 46.5	.707	156.5 ± 44.9	161.7 ± 44.9	155.5 ± 43.0	157.7 ± 45.0	.182
HDL‐C (mg/dL)	42.22 ± 10.8	41.5 ± 9.3	41.3 ± 10.5	42.0 ± 12.5	.596	41.5 ± 11.0	41.2 ± 10.8	42.7 ± 11.0	41.5 ± 10.7	.174
LDL (mg/dL)	85.1 ± 37.6	87.4 ± 31.4	85.8 ± 33.3	87.1 ± 31.5	.716	84.7 ± 31.2	88.2 ± 31.2	85.4 ± 38.2	87.3 ± 31.9	.381
TG (mg/dL)	156.8 ± 97.3	158.7 ± 76.9	158.0 ± 79.8	155.4 ± 80.3	.936	158.6 ± 84.3	167.5 ± 95.0	147.5 ± 65.1	156.3 ± 86.2	.005

*Note*: Data are presented as mean ± SD or *n* (%).

Abbreviations: BMI, body mass index; CVD, cardiovascular disease; DBP, diastolic blood pressure; FBS, fasting blood sugar; HDL, high‐density lipoprotein cholesterol; LDL, low‐density lipoprotein cholesterol; SBP, systolic blood pressure; TC, total cholesterol; TG, triglyceride; WC, waist circumference.

^a^ Analysis of variance was used for continues variables and chi‐square test for categorical variables.

Dietary intakes of contributors according to the quartile of fermented and nonfermented dairy products are summarized in Table 2. A higher fermented dairy product intake was significantly associated with higher intakes of energy, total dairy, fruits and vegetables, proteins, total fat, calcium, and potassium. In contrary, dietary intake of carbohydrate and fiber was higher in the first quartile. Regarding nonfermented dairy product consumption, those in the top quartile had greater intake of total dairy, fruits and vegetables, proteins, fiber, calcium, and potassium, whereas energy and carbohydrate intakes were higher in the bottom quartile.

**TABLE 2:** Dietary intakes of participants according to the quartile of fermented and nonfermented dairy products intake.

Intake quartile (range, g/day)	Fermented dairy products				*p* ^a^	Nonfermented dairy products				*p* ^a^
	Q1 (<397.0)	Q2 (397.0, 512.3)	Q3 (513.1, 653.9)	Q4 (>654.7)		Q1 (≤2.0)	Q2 (2.0, 16.6)	Q3 (16.6, 67.7)	Q4 (≥68.3)	
Energy (kcal/day)	1758.8 ± 32.3	1894.1 ± 29.9	2085.6 ± 28.4	2312.6 ± 28.7	<.001	2653.1 ± 26.1	1761.6 ± 26.2	1663.0 ± 25.4	2055.2 ± 25.4	<.001
Total dairy	118.2 ± 6.5	231.5 ± 6.0	357.8 ± 5.6	595.5 ± 5.8	<.001	204.3 ± 10.3	327.0 ± 9.3	360.2 ± 9.3	461.5 ± 8.8	<.001
Fermented dairy	343.2 ± 4.3	459.3 ± 3.9	574.9 ± 3.7	809.8 ± 3.9	<.001	494.9 ± 9.7	592.5 ± 8.8	601.3 ± 8.7	552.6 ± 8.3	<.001
Nonfermented dairy	50.9 ± 4.7	48.1 ± 4.3	58.8 ± 4.1	61.6 ± 4.2	.101	−14.6 ± 2.7	10.4 ± 2.4	34.9 ± 2.4	184.8 ± 2.3	<.001
Fruits and vegetables	428.6 ± 10.9	419.4 ± 10.0	450.8 ± 9.4	504.8 ± 9.7	<.001	411.7 ± 11.5	458.9 ± 10.4	443.3 ± 10.3	496.5 ± 9.7	<.001
Nutrients (g/day)										
Proteins	74.8 ± 0.8	77.5 ± 0.7	82.2 ± 0.7	88.8 ± 0.7	<.001	76.1 ± 0.9	81.0 ± 0.8	82.1 ± 0.8	85.5 ± 0.7	<.001
Total fat	78.9 ± 1.1	80.2 ± 1.0	81.9 ± 1.0	84.2 ± 1.0	.003	81.1 ± 1.2	82.9 ± 1.1	81.5 ± 1.1	80.5 ± 1.0	.415
Carbohydrate	277.2 ± 2.3	271.5 ± 2.1	262.5 ± 2.0	252.4 ± 2.1	<.001	273.1 ± 2.5	261.8 ± 2.3	263.3 ± 2.2	262.4 ± 2.1	.004
Cholesterol	279.8 ± 6.3	290.5 ± 5.7	294.2 ± 5.4	292.8 ± 5.6	.331	281.8 ± 6.5	286.4 ± 5.9	297.4 ± 5.9	293.3 ± 5.6	.287
Fiber	21.9 ± 0.3	21.5 ± 0.3	20.7 ± 0.3	20.0 ± 0.3	<.001	20.9 ± 0.3	20.9 ± 0.3	20.5 ± 0.3	21.6 ± 0.3	.042
Sodium	2084.1 ± 34.1	2059.9 ± 31.2	2030.5 ± 29.5	1998.7 ± 30.5	.286	2083.9 ± 35.8	2057.2 ± 32.3	2030.6 ± 32.1	1993.3 ± 30.4	.225
Calcium intake	655.1 ± 10.5	822.1 ± 9.7	1011.2 ± 9.1	1367.4 ± 9.4	<.001	792.5 ± 16.4	980.9 ± 14.8	1028.8 ± 14.7	1129.3 ± 13.9	<.001
Potassium intake	2997.2 ± 41.0	3221.4 ± 37.5	3460.1 ± 35.4	3984.2 ± 36.6	<.001	3096.5 ± 45.2	3427.4 ± 40.9	3464.9 ± 40.6	3772.5 ± 38.5	<.001

*Note*: Data are presented as mean ± SE.

^a^ Obtained from ANCOVA (all dietary intakes were adjusted for total energy intake, age, and sex).

Mean intake of dairy foods based on their fat content and fermentation status are presented in Table 3. Mean total dairy products intake was 339.8 ± 223.52 g/day. Total dairy intake of 85.4% was from fermented dairy products and the remaining 15% was from nonfermented dairy products. Mean intake of high‐fat and low‐fat fermented dairy products was 72.1 and 213.3 g/day, respectively. The corresponding values for nonfermented dairy products were 1.2 and 53.2 g/day, respectively.

**TABLE 3:** Mean intake of dairy foods based on their fat content and fermentation status (g/day).

	Mean	SD	Proportion of total dairy product intake (%)
Dairy (g/day)	339.8	223.52	**–**
Fermented dairy products (g/day)	285.4	194.8	84.0
High‐fat fermented dairy products (g/day)	72.1	108.8	21.2
Cheese	8.9	16.7	2.6
Yogurt	61.2	101.9	18.0
Curd	2.0	4.8	0.6
Low‐fat fermented dairy products (g/day)	213.3	159.1	62.8
Dough (yogurt drink)	102.4	113.1	30.1
Yogurt	93.2	103.8	27.4
Cheese	17.7	18.4	5.2
Nonfermented dairy products (g/day)	54.5	92.9	16.0
Low‐fat nonfermented dairy products (<3.5% fat)	53.2	92.9	15.6
Low‐fat milk	15.6	51.2	4.6
Regular‐fat milk	37.6	77.2	11.1
High‐fat nonfermented dairy products (≥3.5% fat)			
High‐fat milk	1.2	3.0	0.3

*Note*: Data are presented as mean ± SD or *n* (%).

Table 4 illustrates crude and multivariable adjusted odds ratios (OR) and 95% confidence intervals (CI) for HTN across the quartiles of dairy products. No insignificant association was observed between total dairy and nonfermented dairy products intake and the odds of HTN either in the crude or in any of the adjusted models. In terms of fermented dairy products, in the crude model, participants in the fourth quartile tend to have lower risk for HTN in comparison with the first quartile (OR = 0.70, 95% CI: 0.52, 0.96; *p* for trend = .058). However, after controlling for possible confounders, the significance disappeared.

**TABLE 4:** Crude and multivariable‐adjusted odds ratios (OR) and 95% confidence intervals (CI) for HTN across the quartiles of dairy products.

	Intake quartile				*p* trend^a^
	Q1	Q2	Q3	Q4	
Total dairy products					
Median intake (IQR) (g/day)	124.46 (76.62, 161.17)	251.39 (220.62, 277.69)	370.63 (338.22, 409.14)	571.84 (503.12, 700.43)	
Events/participants (*n*)	112/463	109/464	112/464	102/463	
Crude model	1	0.96 (0.71, 1.30)	0.997 (0.74, 1.35)	0.88 (0.65, 1.20)	.508
Model 1	1	0.91 (0.67, 1.23)	0.91 (0.67, 1.24)	0.84 (0.62, 1.15)	.306
Model 2	1	0.95 (0.69, 1.30)	0.96 (0.70, 1.31)	0.85 (0.62, 1.18)	.242
Model 3	1	0.96 (0.69, 1.32)	1.01 (0.73, 1.39)	0.90 (0.65, 1.24)	.405
Fermented dairy products					
Median intake (IQR) (g/day)	335.34 (309.48, 367.71)	465.34 (418.06, 490.68)	570.91 (543.95, 604.31)	769.82 (710.23, 872.42)	
Events/participants (*n*)	106/392	103/456	123/508	103/498	
Crude model	1	0.79 (0.58, 1.08)	0.86 (0.64, 1.16)	0.70 (0.52, 0.96)	.058
Model 1	1	0.80 (0.58, 1.10)	0.92 (0.67, 1.26)	0.79 (0.57, 1.10)	.298
Model 2	1	0.81 (0.58, 1.12)	1.01 (0.73, 1.39)	0.81 (0.58, 1.14)	.232
Model 3	1	0.80 (0.57, 1.12)	1.03 (0.74, 1.43)	0.83 (0.59, 1.17)	.263
Nonfermented dairy products					
Median intake (IQR) (g/day)	−9.93 (−18.48, −0.92)	9.38 (4.91, 13.48)	27.37 (19.46, 44.75)	179.62 (107.49, 222.20)	
Events/participants (*n*)	85/449	117/476	113/440	120/489	
Crude model	1	1.10 (0.80, 1.52)	1.42 (1.05, 1.93)	1.20 (0.88, 1.64)	.107
Model 1	1	0.87 (0.60, 1.26)	1.05 (0.72, 1.52)	0.996 (0.71, 1.40)	.674
Model 2	1	0.83 (0.57, 1.22)	1.03 (0.70, 1.51)	0.93 (0.66, 1.32)	.642
Model 3	1	0.83 (0.57, 1.23)	1.08 (0.73, 1.59)	0.99 (0.69, 1.41)	.413

*Note*: Model 1: Adjusted for age (years), sex (male/female), daily energy intake (kcal/day). Model 2: Additionally, adjusted for smoking (yes/no), level of education (under graduate/graduate/postgraduate), ethnicity, and marital status (single/married/divorce or widow). Model 3: Additionally, adjusted for body mass index (kg/m^2^).

^a^ Derived from a Mantel–Haenszel extension chi‐square test.

Table 5 indicates the OR and 95% CIs for HTN across the quartiles of low‐fat and high‐fat fermented dairy products. Low‐fat fermented dairy products intake was not pertinent to the risk of HTN either in the crude or in adjusted models. Regarding high‐fat fermented dairy products, in the crude model, those in the highest quartile had 34% lower risk for HTN compared to those in the first quartile (OR = 0.66, 95% CI: 0.49, 0.88; *p* for trend < .001). Adjustment for potential risk factors weakened the association but remained statistically significant (OR = 0.73, 95% CI: 0.53, 1.01; *p* for trend = .001). In terms of low‐fat nonfermented dairy products, those with the greatest intake were at a higher risk for HTN (OR = 1.39, 95% CI: 1.02, 1.90; *p* for trend = .040) in the crude model. However, after adjustment for potential confounders, this association disappeared. No significant association was found between high‐fat nonfermented dairy products and the odds of HTN in different models.

**TABLE 5:** Crude and multivariable‐adjusted odds ratios (OR) and 95% CIs for HTN across the quartiles of low‐fat and high‐fat dairy products.

	Intake quartile				*p* trend^a^
	Q1	Q2	Q3	Q4	
Fermented dairy products					
Low‐fat fermented dairy products					
Median intake (IQR) (g/day)	238.83 (222.40, 264.02)	331.69 (305.97, 363.12)	440.26 (419.55, 466.68)	608.19 (530.44, 677.65)	
Events/participants (*n*)	94/416	122/454	113/502	106/482	
Crude model	1	1.26 (0.92, 1.72)	0.99 (0.73, 1.36)	0.97 (0.70, 1.32)	.445
Model 1	1	1.20 (0.88, 1.65)	1.01 (0.74, 1.39)	1.05 (0.76, 1.46)	.919
Model 2	1	1.23 (0.89, 1.71)	0.96 (0.69, 1.34)	1.06 (0.75, 1.49)	.798
Model 3	1	1.26 (0.91, 1.76)	0.99 (0.71, 1.38)	1.08 (0.76, 1.53)	.825
High‐fat fermented dairy products					
Median intake (IQR) (g/day)	67.67 (67.67, 67.67)	69.10 (69.00, 71.95)	113.38 (97.67, 154.90)	286.24 (200.53, 317.67)	
Events/participants (*n*)	143/496	114/423	73/437	105/498	
Crude model	1	0.91 (0.68, 1.22)	0.49 (0.36, 0.68)	0.66 (0.49, 0.88)	<.001
Model 1	1	0.88 (0.65, 1.18)	0.49 (0.35, 0.67)	0.68 (0.50, 0.92)	<.001
Model 2	1	0.88 (0.65, 1.18)	0.55 (0.39, 0.77)	0.71 (0.52, 0.98)	<.001
Model 3	1	0.91 (0.66, 1.23)	0.57 (0.40, 0.81)	0.73 (0.53, 1.01)	.001
Cheese					
Median intake (IQR) (g/day)	8.68 (4.33, 12.18)	19.22 (16.82, 23.15)	29.54 (27.76, 31.20)	43.10 (34.81, 58.59)	
Events/participants (*n*)	99/463	133/464	98/464	105/463	
Crude model	1	1.48 (1.09, 1.99)	0.98 (0.72, 1.35)	1.08 (0.79, 1.47)	.677
Model 1	1	1.47 (1.09, 1.99)	0.92 (0.67, 1.27)	0.99 (0.72, 1.36)	.309
Model 2	1	1.50 (1.10, 2.06)	0.96 (0.69, 1.34)	1.17 (0.84, 1.63)	.296
Model 3	1	1.47 (1.07, 2.01)	0.95 (0.68, 1.32)	1.10 (0.78, 1.53)	.180
Yogurt and dough					
Median intake (IQR) (g/day)	64.70 (25.98, 95.61)	178.86 (154.62, 206.86)	284.43 (258.05, 311.66)	467.61 (401.98, 564.75)	
Events/participants (*n*)	111/463	107/464	115/464	102/463	
Crude model	1	0.95 (0.70, 1.29)	1.04 (0.77, 1.41)	0.90 (0.66, 1.22)	.641
Model 1	1	0.88 (0.65, 1.21)	0.99 (0.73, 1.34)	0.86 (0.63, 1.17)	.504
Model 2	1	0.93 (0.67, 1.27)	1.03 (0.75, 1.41)	0.86 (0.62, 1.19)	.404
Model 3	1	0.94 (0.68, 1.29)	1.06 (0.77, 1.45)	0.89 (0.64, 1.23)	.497
Nonfermented dairy products					
Low‐fat nonfermented dairy products					
Median intake (IQR) (g/day)	−11.21 (−18.77, −4.76)	8.26 (4.54, 11.79)	26.15 (18.69, 42.21)	175.92 (102.52, 217.57)	
Events/participants (*n*)	86/455	115/472	114/437	120/490	
Crude model	1	1.38 (1.01, 1.89)	1.51 (1.10, 2.08)	1.39 (1.02, 1.90)	.040
Model 1	1	1.12 (0.76, 1.64)	1.15 (0.77, 1.72)	1.17 (0.83, 1.65)	.421
Model 2	1	1.08 (0.73, 1.60)	1.14 (0.76, 1.73)	1.11 (0.78, 1.60)	.394
Model 3	1	1.08 (0.73, 1.61)	1.14 (0.75, 1.72)	1.16 (0.81, 1.67)	.276
High‐fat nonfermented dairy products					
Median intake (IQR) (g/day)	−0.42 (−0.73, −0.19)	0.33 (0.20, 0.45)	0.81 (0.70, 0.94)	3.10 (1.83, 5.33)	
Events/participants (*n*)	91/463	121/464	131/464	92/463	
Crude model	1	1.44 (1.06, 1.96)	1.61 (1.18, 2.18)	1.01 (0.73, 1.40)	.750
Model 1	1	1.25 (0.86, 1.82)	1.24 (0.81, 1.91)	0.91 (0.63, 1.30)	.266
Model 2	1	1.16 (0.79, 1.71)	1.10 (0.71, 1.71)	0.75 (0.52, 1.10)	.270
Model 3	1	1.15 (0.78, 1.70)	1.05 (0.67, 1.64)	0.76 (0.52, 1.11)	.258

*Note*: Model 1: Adjusted for age (years), sex (male/female), daily energy intake (kcal/day). Model 2: Additionally, adjusted for smoking (yes/no), level of education (under graduate/graduate/postgraduate), ethnicity, and marital status (single/married/divorce or widow). Model 3: Additionally, adjusted for body mass index (kg/m^2^).

^a^ Derived from a Mantel–Haenszel extension chi‐square test.

The OR and 95% CIs for HTN across the quartiles of dairy products in different ethnicities are highlighted in Table 6. Consistently across all ethnicities, similar to the whole population, no significant association was found between the consumption of total, fermented, and nonfermented dairy products and HTN.

**TABLE 6:** Crude and multivariable‐adjusted odds ratios (OR) and 95% confidence intervals (CI) for HTN across the quartiles of dairy products in different ethnicity across.

	Total dairy products				*p* trend^a^	Fermented dairy products				*p* trend^a^	Nonfermented dairy products				*p* trend^a^
	Q1	Q2	Q3	Q4		Q1	Q2	Q3	Q4		Q1	Q2	Q3	Q4	
Fars															
Event/participants	72/265	60/268	70/261	61/239		74/250	60/275	65/255	64/253		45/203	70/273	73/275	75/282	
Crude model	1	0.77 (0.52, 1.15)	0.98 (0.67, 1.44)	0.92 (0.62, 1.37)	.965	1	0.66 (0.45, 0.98)	0.81 (0.55, 1.20)	0.80 (0.54, 1.19)	.474	1	1.21 (0.79, 1.86)	1.27 (0.73, 1.94)	1.27 (0.73, 1.94)	.290
Model 1	1	0.72 (0.48, 1.08)	0.88 (0.59, 1.30)	0.84 (0.56, 1.26)	.611	1	0.66 (0.44, 0.98)	0.82 (0.55, 1.24)	0.84 (0.55, 1.28)	.636	1	0.99 (0.60, 1.66)	0.99 (0.59, 1.67)	1.04 (0.65, 1.68)	.820
Azari															
Event/participants	10/58	14/44	9/33	4/35		3/32	11/46	20/62	3/30		15/52	11/50	4/30	7/38	
Crude model	1	2.24 (0.88, 5.68)	1.80 (0.65, 5.02)	0.62 (0.18, 2.15)	.648	1	3.04 (0.77, 11.9)	4.60 (1.25, 16.93)	1.07 (0.20, 5.79)	.522	1	0.70 (0.28, 1.71)	0.38 (0.11, 1.27)	0.56 (0.20, 1.54)	.152
Model 1	1	2.72 (1.03, 7.22)	2.38 (0.81, 7.05)	0.74 (0.21, 2.65)	.900	1	2.72 (0.67, 10.97)	4.17 (1.12, 15.53)	0.82 (0.15, 4.60)	.759	1	1.11 (0.36, 3.42)	0.49 (0.13, 1.80)	0.76 (0.24, 2.39)	.371
Kord															
Event/participants	7/39	4/45	5/55	4/42		6/25	4/41	6/58	4/57		3/66	7/49	6/30	4/36	
Crude model	1	0.45 (0.12, 1.66)	0.46 (0.13, 1.56)	0.48 (0.13, 1.79)	.266	1	0.34 (0.09, 1.36)	0.36 (0.11, 1.27)	0.24 (0.06, 0.94)	.062	1	3.50 (0.86, 14.30)	5.25 (1.21, 22.68)	2.62 (0.55, 12.44)	.147
Model 1	1	0.38 (0.10, 1.52)	0.37 (0.10, 1.39)	0.33 (0.08, 1.32)	.123	1	0.31 (0.07, 1.31)	0.48 (0.13, 1.81)	0.32 (0.07, 1.37)	.221	1	1.35 (0.24, 7.77)	1.86 (0.25, 13.78)	1.39 (0.24, 8.25)	.734
Gilak															
Event/participants	3/31	2/38	2/42	1/47		3/28	3/29	0	2/50		3/38	1/34	1/45	3/41	
Crude model	1	0.52 (0.08, 3.32)	0.47 (0.07, 2.98)	0.20 (0.02, 2.05)	.166	1	0.96 (0.18, 5.22)	0.00 (0.00, –)	0.347 (0.054, 2.22)	.091	1	0.35 (0.03, 3.57)	0.26 (0.03, 2.66)	0.92 (0.17, 4.87)	.869
Model 1	1	0.53 (0.08, 3.47)	0.53 (0.08, 3.48)	0.23 (0.02, 2.33)	.215	1	0.95 (0.17, 5.35)	0.00 (0.00, –)	0.303 (0.04, 2.30)	.082	1	0.21 (0.01, 3.18)	0.12 (0.01, 2.29)	0.63 (0.09, 4.46)	.925
Bakhtiari and Lor															
Event/participants	17/55	20/42	17/50	23/69		15/44	19/42	22/56	21/74		16/61	14/50	24/40	23/65	
Crude model	1	2.03 (0.88, 4.67)	1.15 (0.51, 2.61)	1.12 (0.52, 2.39)	.865	1	1.60 (0.67, 3.81)	1.25 (0.55, 2.85)	0.77 (0.34, 1.71)	.314	1	1.09 (0.47, 2.53)	4.22 (1.80, 9.89)	1.54 (0.72, 3.31)	.072
Model 1	1	2.29 (0.91, 5.79)	1.02 (0.42, 2.50)	1.30 (0.57, 2.97)	.964	1	3.04 (1.12, 8.27)	1.88 (0.76, 4.70)	1.58 (0.63, 3.98)	.708	1	0.59 (0.22, 1.59)	1.57 (0.54, 4.57)	0.95 (0.41, 2.20)	.643
Balouch and Arab and Qashqaei															
Event/participants	3/14	9/26	9/22	9/30		5/12	6/23	10/23	9/34		4/24	9/25	11/20	6/23	
Crude model	1	1.94 (0.43, 8.79)	2.54 (0.55, 11.77)	1.57 (0.35, 7.02)	.705	1	0.49 (0.11, 2.16)	1.08 (0.26, 4.42)	0.50 (0.13, 1.99)	.563	1	2.81 (0.73, 10.84)	6.11 (1.52, 24.50)	1.76 (0.43, 7.31)	.297
Model 1	1	1.29 (0.26, 6.37)	2.06 (0.42, 10.24)	1.06 (0.22, 5.19)	.963	1	0.41 (0.08, 2.02)	1.32 (0.28, 6.16)	0.57 (0.12, 2.72)	.892	1	3.85 (0.55, 26.72)	7.26 (0.84, 62.71)	1.80 (0.34, 9.59)	.870

*Note*: Model 1: Adjusted for age (years), sex (male/female), and daily energy intake (kcal/day).

^a^ Derived from a Mantel–Haenszel extension chi‐square test.

## 4 DISCUSSION

In this cross‐sectional study, we assessed whether total dairy product consumption or its various subcategories were related to the risk of HTN in PCAD patients from the IPAD study. Total dairy and nonfermented dairy products consumption had no association with HTN risk, but fermented dairy products had a negative correlation with HTN in the crude model, whereas after controlling for possible confounders, the significance disappeared. Additional analyses of low‐fat and high‐fat categories indicated that the intake of low‐fat fermented and high‐fat nonfermented dairy products intake was not related to the risk of HTN. However, the higher the intake of high‐fat fermented dairy products, the lower the risk for HTN.

Several prior studies have examined the correlation between dairy consumption and HTN, however, the findings have been inconclusive (Alonso et al., 2005; Koskinen et al., 2018; Mirmiran et al., 2016). A prospective cohort study on aged US women with a mean 10 years of follow‐up showed an inverse association between total dairy and low‐fat dairy consumption and the risk of HTN (Wang et al., 2008). In a 27‐month study called Seguimiento Universidad de Navarra, it was found that people who consumed low‐fat dairy products had a lower risk of developing HTN. However, they found no significant link between consuming whole‐fat dairy products and developing HTN (Alonso et al., 2005). Additionally, a cross‐sectional study of Chinese adults found that those who consumed dairy products at least once a week had a lower risk of HTN than those who consumed dairy products less than once a week (Wang et al., 2022). In a meta‐analysis of 16 cohort studies, Heidari et al. suggested a reduction in the HTN incidence risk by 10%, 14%, and 5% for the consumption of total, low‐fat, and fermented dairy products, respectively. Nevertheless, they showed that these findings were dependent on sex and geographical region of the study (Heidari et al., 2021). In line with the findings of this study, no association was found between total dairy and HTN in the general Dutch population study (Engberink et al., 2009). Similarly, a prospective cohort study on 4304 participants revealed no association between elevated BP and dairy intake (Steffen et al., 2005). Moreover, the results of a cohort study showed no association between total, low‐fat, full‐fat, and fermented dairy intake and incident HTN over a period of 10 years, on 1750 middle‐aged individuals from the United Kingdom (Heraclides et al., 2012).

Moreover, a cross‐sectional study on 29,378 Iranian individuals revealed no association between dairy and vegetable intakes and HTN (Nouri et al., 2023).

Our findings indicate that the process of fermentation could potentially influence the relationship between dairy products and the likelihood of developing HTN. As the intake of high‐fat fermented dairy products increased, the risk of HTN decreased in this study, but no significant association was found between high‐fat nonfermented dairy products and the odds of HTN. In a cross‐sectional study on 5616 Iranian adults, higher intake of whole‐fat dairy was related with 24% lower risk of HTN (Mirmiran et al., 2015). In addition, the results of the Prospective Urban Rural Epidemiology (PURE) study on 147,812 individuals from 21 countries, with a median follow‐up of 9.1 years, revealed that higher whole‐fat dairy intake was significantly associated with a lower incidence of HTN (Bhavadharini et al., 2020). Moreover, a Mendelian randomized study reported the protective effect of semiskimmed milk against essential HTN, whereas skim milk had the opposite effect (Shi et al., 2023). A meta‐analysis on the effect of dairy consumption and metabolic syndrome and its components declared that higher whole‐fat dairy intake was associated with 22% lower risk of metabolic syndrome (Lee et al., 2018). It is well established that metabolic syndrome contributes to the development of HTN through the accumulation of visceral adipose tissue and its adipocytokines (Yanai et al., 2008). Contrary to our finding, several studies found a positive or neutral relation between high‐fat dairy and HTN (Schmidt et al., 2021; Soedamah‐Muthu et al., 2012; Toledo et al., 2008). They claimed that high‐fat dairy products have higher amounts of saturated fatty acids (SFAs) and are related to more weight gain and addition energy intake and therefore whole‐fat dairy products can cause HTN (Alonso et al., 2009). Although SFA of high‐fat dairy products is a possible mechanism for HTN and cardiometabolic events, not all evidence supports this and there is still inconclusive findings (Krauss & Kris‐Etherton, 2020). For instance, some studies have even reported a beneficial association between SFA and HTN (Currenti et al., 2022; Nakamura et al., 2019). The NutriNet‐Santé cohort study revealed that despite being a notable source of dietary saturated fats, dairy consumption was not associated with cardiovascular risk (Deschasaux‐Tanguy et al., 2022).

Discrepancies in terms of the SFA–HTN relationship can be due to differences in the manufacturing process, such as fermentation. Fermentation process includes adding viable bacteria to dairy foods. One of the most fundamental changes during fermentation by lactic acid bacteria is hydrolysis of some milk proteins and producing several peptides and free amino acids which can have BP‐lowering effects (Takano, 2002). Valine–proline–proline and isoleucine–proline–proline are two of these peptides by ACE inhibitory effect in vitro and are assumed to lower BP through this mechanism (Xu et al., 2008). In addition, based on the type of probiotic used in fermentation, some dairy products contain γ‐aminobutyric acid (Galli et al., 2022), which could effectively reduce BP after 12 weeks of daily intake in mildly hypertensive patients (Inoue et al., 2003). Moreover, living microorganism contents of fermented dairy products can improve gut microbiota composition and functionality (González et al., 2019). A recent review claimed that, consumption of more complex dairy products especially fermented forms like yogurt, seem to be inversely related to outcomes of cardiovascular disease (Nestel & Mori, 2023). In our study, high‐fat fermented dairy was linked to lower HTN risk, while high‐fat nonfermented dairy showed no association that is possibly owing to its low amount of consumption in our study population. According to the FAO reports, Iran is among countries with low milk consumption per capita (<30 kg/capita/year; The Food and Agriculture Organization of the United Nations), and compared to other studies (Koskinen et al., 2018), the average intake of nonfermented dairy among Iranians is much lower.

It is better to note that, discrepancies between the studies may be related to more than mentioned mechanisms, contrary to our findings, some studies declared that dairy products can increase the risk of HTN due to their advanced glycation end products (AGEs) contents. AGEs, which are formed as a result of the Maillard reaction during the processing of milk and dairy products, have the potential to cause arterial stiffness (Dong et al., 2023).

No significant association was found between low‐fat fermented dairy products and HTN, though their consumption exceeded that of high‐fat dairy products. This could be due to the consumed proportion of various dairy products in fermented or nonfermented dairy foods. For example, dough (an Iranian drink based on yogurt) and cheese accounted for over 50% of total low‐fat fermented dairy products. Dough and cheese are high in sodium and it is possible that this amount of sodium counteracts the BP lowering effect of dairy bioactive peptides (Soedamah‐Muthu et al., 2012).

This study has some substantial strengths. First, this study is the initial investigation that explored the correlation between fermented and nonfermented dairy products and HTN in PCAD participants. Second, conducting subgroup analyses according to the amount of fat content of dairy products helped us to explore the effect of fat content of dairy products on HTN. By using a large and diverse sample size from different regions and ethnic backgrounds, our research outcomes are more externally valid. This means that our findings can be applied to various Iranian populations, making our results more generalizable. This research is accompanied by some constraints. First, the nature of cross‐sectional studies prevented the creation of a cause‐and‐effect relationship between fermented and nonfermented dairy products and HTN. Furthermore, by employing FFQ, recall bias and misclassification of individuals within the quartiles of fermented and nonfermented dairy products are probable in this study. Third, although various confounders were taken into account in our statistical analysis, residual confounding owing to unknown or unmeasured factors such as sodium may affect our results.

## 5 CONCLUSION

In conclusion, we found that moderate consumption of high‐fat fermented dairy products, in a population with low consumption of dairy products, has beneficial effects on reducing the risk of HTN. Future clinical trials are warranted to explore the effect of various types of dairy products on BP. Furthermore, it is recommended to measure the amounts of ACE inhibitory peptides in fermented and nonfermented dairy products to assess the effect of fermentation in this respect.

## AUTHOR CONTRIBUTIONS

**Shakila Ansari:** Conceptualization (equal); writing – original draft (equal); writing – review and editing (equal). **Noushin Mohammadifard:** Data curation (lead); project administration (equal); writing – review and editing (equal). **Parisa Hajihashemi:** Writing – original draft (equal). **Fahimeh Haghighatdoost:** Conceptualization (equal); supervision (lead); writing – review and editing (equal). **Ehsan Zarepur:** Resources (equal). **Shirin Mahmoudi:** Formal analysis (lead). **Fatemeh Nouri:** Data curation (equal). **Fereydoon Nouhi:** Resources (equal). **Tooba Kazemi:** Resources (equal). **Nahid Salehi:** Resources (equal). **Kamal Solati:** Resources (equal). **Samad Ghaffari:** Resources (equal). **Mahboobeh Gholipour:** Resources (equal). **Mostafa Dehghani:** Resources (equal). **Mostafa Cheraghi:** Resources (equal). **Habib Heybar:** Resources (equal). **Hassan Alikhasi:** Resources (equal). **Nizal Sarrafzadegan:** Funding acquisition (lead); project administration (equal); resources (equal).

## CONFLICT OF INTEREST STATEMENT

None of the authors had any personal or financial conflicts of interest.

## FUNDING INFORMATION

This study has been funded by the Research and Technology Department, Iran Ministry of Health and Medical Education and the Iranian Network of Cardiovascular Research with a grant number of 96110 and the Isfahan University of Medical Sciences.

## ETHICS STATEMENT

This study was performed based on the guidelines laid down in the Declaration of Helsinki.

## CONSENT TO PARTICIPATE

A written informed consent form was taken from all participants before entering the study.

## CONSENT FOR PUBLICATION

Not applicable.

## Data Availability

The data that support the findings of this study are available from the corresponding author upon reasonable request.
